# Annotations

**Published:** 1909-12-25

**Authors:** 


### ANNOTATIONS.

### The Beit Research Fellowships.

THE £215,000 which Mr. Otto Beit and his brother, the late Mr. Alfred Beit, have given to found the "Beit Memorial Fellowships for Medical Research" will place medical research on a worthy footing. The 30 Fellowships eventually to be filled by it are a statesmanlike way of co-ordinating in some measure the work of individual investigators, which has been too often in the past sporadic, overlapping, and wasteful. From the fact that these Fellowships may be held by "any man or woman of European descent, graduate of any approved University within the British Empire," medical research is raised, as it has needed raising, to the position of an imperial policy. The justification of an empire is the co-ordination of its parts for the common advantage, and there is no part of the Empire which has not its own problems and which medical research cannot benefit. This is why we congratulate the founder on admitting all branches of medical research work to the benefits of his great scheme. The £250 a year for three years which the Fellowship bestows is practical on grounds both of its value and its length of tenure, and the University of London may congratulate itself on having received notice of Mr. Beit's gift and of having as one of the trustees of the fund its Principal. The first election of Fellows (to number not more than 10 on that occasion) will take place on March 1, 1910. We believe that this endowment of research as a whole will do far more for medicine and science than special gifts to particular objects of research have been able to hope for.

### Popular Fetishes.

EVERY practitioner is familiar with the multitude of popular superstitions that are to be encountered among the poorer classes in this country. From time to time some sympathetic soul writes a paper on these interesting fads and fancies, and the subject, indeed, is one that admits of closer study and attention than most of us can give to it. The latest contribution to the literature is Dr. Eleanor Lowry's address to the Birmingham Branch of the National Union of Women Workers. Dr. Lowry is medical inspector of schools at Wimbledon, and her experiences have shown her that not the least of the forces that work against her is popular superstition. Thirty per cent. of the children she inspected wear necklaces of blue beads or cheap coral as prophylactics against common colds and quinseys. She found boys with the dirty string necklace common enough, and worn for the same reason, and in one case she records a new treatment, popularly said to be highly efficacious, for running ears. This consists in poulticing the affected ear with a quid of chewing tobacco —and trusting to luck. The practice of piercing the ears to improve the eyesight is one with which most doctors have had a tilt, and there is little doubt that the average slum child is brought up to believe in charms and amulets of a nature that would shock a self-respecting South Sea Islander. Medical folk lore is an enthralling subject for discussion and speculation, and we commend it to the general practitioner who has the time or the inclination to compile a paper, somewhat on the same lines as Dr. Lowry's, dealing with these popular fetishes. Ignorance and apathy are the two great giants which all doctors have to fight against in their struggle to inculcate into their poorer patients the ordinary essentials of hygiene. Such compilations as we suggest will enable those of us who are waging that struggle to understand the motives and underlying causes of many of these popular superstitions and practices, and therefore to be in a position to cope with them more effectually.

### Forcible Feeding in Prisons.

No verdict such as was given last week in the Lord Chief Justice's Court, in the case wherein certain mischievous women sued the Home Secretary and a prison doctor for assault consequent upon an attempt to feed the plaintiffs by means of a nasal tube, was needed to justify the action of the prison authorities in the eyes of the medical profession, or indeed in the eyes of every thoughtful, sensible person. We have no desire to comment on such "expert medical evidence" as was given on behalf of the plaintiffs, but it may be well to point out that this case is no illustration of the truth of the old adage anent the disagreement of doctors. The faculty as a whole subscribes to every word that was said by the medical witnesses for the defence, expert witnesses who, it is safe to say, have had a far wider and more thorough experience in the methods of forced feeding than any of the witnesses for the plaintiffs. Every agitation, such as that so foolishly engineered by some women of the plaintiffs' class, will find supporters even among medical men, and it is only natural that in many cases a sense of indignation, whether virtuous or otherwise, should overcome the logical sense and blind partisans to the real facts and the true issues of the subject. The verdict in this action has made it clear that a prison doctor is fully entitled to take such measures, even amounting to technical assault, as may help him to improve the health or prevent the physical deterioration of prisoners. That is a conclusion which has always been tacitly understood, but, so far as we are aware, it has never been laid down as a law rule, and the present action will therefore be a useful precedent to cite in future cases. It is conceivable that a prisoner, determined to be a martyr, may object to a necessary and life-saving operation. In a hospital the surgeon has no right to insist upon doing the operation, but in prison the medical attendant would be perfectly justified in disregarding the patient's expressed wishes and proceeding with the operation so long as such action is absolutely essential and in the interests of the prisoner. That is the rule laid down by the Chief Justice, and it appears to be a very clear, sensible, and straightforward exposition of the law.

